# Therapeutic drug monitoring of ganciclovir for postnatal cytomegalovirus infection in an extremely low birth weight infant: a case report

**DOI:** 10.1186/s12887-016-0683-x

**Published:** 2016-08-22

**Authors:** Mariko Sunada, Daisuke Kinoshita, Naoko Furukawa, Minako Kihara, Akira Nishimura, Masako Moriuchi, Hiroyuki Moriuchi

**Affiliations:** 1 Department of Neonatology, Japanese Red Cross Kyoto Daiichi Hospital, 15-749 Honmachi, Higashiyama-ku, Kyoto City, Kyoto 605-0981 Japan; 2 Department of Pediatrics, Nagasaki University Hospital, Graduate School of Biomedical Sciences, Nagasaki University, Nagasaki, Japan

**Keywords:** Ganciclovir, Postnatal cytomegalovirus infection, Therapeutic drug monitoring, Extremely low birth weight infant

## Abstract

**Background:**

Ganciclovir is a therapeutic choice for extremely premature infants with severe postnatal cytomegalovirus disease, but little is known about its optimal dose size and dosing interval for them.

**Case presentation:**

We treated an extremely premature female infant with postnatal cytomegalovirus infection with intravenous administration of ganciclovir since 49 days of life (postmenstrual age of 31 weeks). After ganciclovir treatment was initiated at a dose of 5 mg/kg every 12 h, cytomegalovirus loads in the peripheral blood were markedly decreased. However, since plasma ganciclovir trough level was too high, the interval was extended to every 24 h. Subsequently, the trough level and the estimated 12-h area under the concentration–time curve (AUC_0–12_) were decreased from 3.5 mg/L to 0.3 mg/L and 53.9 mg · h/L to 19.2 mg · h/L, respectively, resulting in an exacerbation of viremia and clinical condition. Adjustment of dosing interval from 24 h to 12 h led to a peak level of 4.2 mg/L, trough level of 1.1 mg/L, and AUC_0–12_ of 31.8 mg · h/L, resulting in a marked suppression of viral load.

**Conclusions:**

Monitoring the therapeutic drug levels and cytomegalovirus loads is useful in obtaining a proper treatment effect and preventing overdosage during ganciclovir therapy in premature infants with postnatal cytomegalovirus infection.

**Electronic supplementary material:**

The online version of this article (doi:10.1186/s12887-016-0683-x) contains supplementary material, which is available to authorized users.

## Background

Postnatal cytomegalovirus (CMV) infection, largely acquired via breastmilk, is usually asymptomatic in full-term infants. However, extremely premature infants appear to have a higher risk of developing symptomatic postnatal CMV infection, characterized by hepatitis, neutropenia, thrombocytopenia, respiratory failure, and sepsis-like syndrome [1, 2].

Although ganciclovir is an anti-CMV drug that has been shown to be effective against severe CMV diseases, little is known about its optimal dose size and dosing interval for extremely premature infants [3].

We have recently experienced a case of an extremely premature infant with severe postnatal CMV infection. During the treatment with ganciclovir, we performed therapeutic drug monitoring (TDM) and measurement of CMV load in the peripheral blood that were useful to obtain a proper therapeutic effect and avoid overdosage.

## Case presentation

A 37-year-old primigravid woman was referred to the emergency department due to premature ablation of a normally implanted placenta. After admission, an extremely low birth weight female infant (birth weight, 532 g; length, 27 cm) was delivered by Cesarean section at 24 + 0 weeks of gestation. Her Apgar scores at 1, 5, and 10 min were 1, 3, and 7, respectively. The umbilical artery pH was 6.91. She was intubated shortly after birth due to respiratory distress, and surfactant was administered. The surgical ligation of a patent ductus arteriosus was performed at 15 days of life. She began receiving hydrocortisone from 16 days of life because of respiratory deterioration. Enteral feeding with fresh breast milk was started on 2 days of life, and the infant was on full feeding by 28 days of life.

A cystic lesion had been identified below the liver during the fetal period, and we diagnosed biliary atresia after her birth. On 34 days of life, cholestatic jaundice developed (direct bilirubin 16.9 mg/dL), and a biliary drainage procedure was performed.

On 43 days of life, she developed progressive abdominal distension and a septic appearance. Laboratory values showed thrombocytopenia (41 × 10^3^/μL) and elevation of C-reactive protein levels (16.2 mg/dL). Empiric antibiotic therapy, using vancomycin and meropenem, and intravenous immunoglobulin therapy were started for clinically suspected sepsis. Serial blood cultures were negative for both aerobic and anaerobic bacterial infections.

In view of thrombocytopenia and necrotizing enterocolitis-like ileus, we performed a CMV screening at the same time. Her urine was positive for CMV DNA, and real-time polymerase chain reaction (PCR) showed a VL of 5.0 × 10^6^ copies/mL in the peripheral blood. The CMV DNA from the umbilical cord and Guthrie card, both of which were collected on 16 days of life, were below the limits of detection. Real-time PCR of her mother’s breast milk showed a CMV load of 3.9 × 10^4^ copies/mL. Although she had received five red packed cell transfusions by 43 days of life, they were all irradiated and leucocyte-depleted. Those results suggested that the breast milk was the most likely source of virus transmission to the patient.

As the thrombocytopenia and necrotizing enterocolitis-like ileus further deteriorated, we decided to begin antiviral therapy with intravenous ganciclovir. Ganciclovir therapy (5 mg/kg per dose every 12 h) was initiated at 49 days of life (postmenstrual age of 31 weeks).

We performed the TDM for ganciclovir three times, using liquid chromatography-tandem mass spectrometry. The estimated 12-h area under the concentration–time curve (AUC_0–12_) was determined using the trapezoidal method. When the drug interval was 24 h, the estimated AUC_0–12_ was corrected for half of the calculated value using the trapezoidal method. Table 1 shows the association between the CMV load and the ganciclovir TDM analysis. At 55 days of life (7^th^ treatment day), we performed the first ganciclovir TDM analysis. The plasma ganciclovir levels at 30 min, 90 min, 6 h, and 11 h after ganciclovir infusion were 5.3 mg/L, 6.0 mg/L, 4.0 mg/L, and 3.5 mg/L, respectively; the estimated AUC_0–12_ was 53.9 mg · h/L. The CMV load decreased from 5.0 × 10^6^ to 5.3 × 10^4^ copies/mL in parallel with the increase of platelet counts. The ganciclovir trough level and the estimated AUC_0–12_ were too high, so we extended the dosing interval from 12 h to 24 h on 62 days of life (14^th^ treatment day). On 70 days of life (22^nd^ treatment day), we performed the second TDM analysis, demonstrating that the plasma peak and trough ganciclovir levels were 6.0 mg/L and 0.3 mg/L, respectively, and the estimated AUC_0–12_ was 19.2 mg · h/L. Concurrently, the CMV load modestly rebounded from 5.3 × 10^4^ to 1.6 × 10^5^ copies/mL, and the thrombocytopenia worsened (35 × 10^3^/μL). Therefore, we shortened the dosing interval from 24 h to 12 h on 29^th^ treatment day. On 84 days of life (36^th^ treatment day), we performed the third TDM analysis, demonstrating that the plasma peak and trough ganciclovir levels were 4.2 mg/L and 1.1 mg/L, respectively, and the estimated AUC_0–12_ was 31.8 mg · h/L. Although the VL markedly decreased from 1.6 × 10^5^ to 2.4 × 10^3^ copies/m/L, her clinical condition deteriorated with thrombocytopenia, hepatitis (aspartate transaminase: 422 IU/L; alanine transaminase: 87 IU/L), and unconjugated hyperbilirubinemia (serum direct bilirubin: 10.3 mg/dL). Neither neutropenia nor renal impairment was observed during ganciclovir therapy. On 85 days of life (37^th^ treatment day), the ganciclovir therapy was discontinued, because of the concern that the thrombocytopenia and hepatitis resulted from its adverse reactions. Her clinical condition further deteriorated with increase of VL to 1.0 × 10^6^ copies/mL on 107 days of life. On 109 days of life, she died of hepatic failure. Extensive hepatocyte necrosis was noted at necropsy and on histopathological examination; however, the precise mechanism of liver injury was not revealed by the postmortem analyses. This infant’s clinical course is summarized in the accompanying supplemental time-line file (Additional file 1).

**Table 1:** Association between CMV viral load and ganciclovir TDM analysis

Days of life	Weeks of postmenstrual age	Weight (g)	Serum creatinine (mg/dl)	Ganciclovir therapy		Ganciclovir TDM			CMV load^d^ (copies/ml)
				Dose (mg/kg)	Interval (hour)	Peak level^a^ (mg/l)	Trough level^b^ (mg/l)	Estimated AUC_0–12_^c^ (mg ▪ h/l)	
48	30 + 6	1132	0.39	Reference (pre-therapy)					5.0 × 10^6^
55	31 + 6	1296	0.32	5	12	6.0	3.5	53.9	5.3 × 10^4^
70	34 + 0	1120	0.26	5	24	6.0	0.3	19.2	1.6 × 10^5^
84	36 + 0	1326	0.27	5	12	4.2	1.1	31.8	2.4 × 10^3^
107	39 + 2	2058	0.32	22 days post-therapy					1.0 × 10^6^

*CMV* cytomegalovirus, *TDM* therapeutic drug monitoring, *AUC* area under the concentration-time curve

^a^The peak levels of ganciclovir were measured at 90 min after ganciclovir infusion

^b^The trough levels of ganciclovir were measured at 11 h after ganciclovir infusion

^c^The estimated values of AUC_0–12_ were calculated using Trapezoidal rule

^d^CMV loads in the peripheral blood were determined by real-time PCR

## Conclusions

To our knowledge, this was the first description of an extremely premature infant with postnatal CMV infection who had multiple ganciclovir TDM and CMV load analyses. No controlled study has yet evaluated the impact of antiviral treatment on the outcome of postnatal CMV infection, and there have been no treatment recommendations yet [3]. In the present case, we adjusted the dosing interval according to the results of the ganciclovir TDM and CMV load. Ganciclovir was effective in the reduction of the CMV load in parallel with temporal improvement of her clinical condition, but unfortunately, our patient died.

There have been very few TDM data of ganciclovir therapy in extremely premature infants [4]. The response to therapy with ganciclovir has been influenced by the interindividual pharmacokinetic variability. Thus, low plasma levels of ganciclovir are associated with therapy failure and also predispose the virus to drug resistance, although optimal therapeutic drug levels have not been well established [4]. Plasma ganciclovir trough levels below 0.6 mg/L are associated with therapy failure during the intravenous ganciclovir administration for CMV retinitis [5]. Based on existing data and concerns regarding under-treatment, a trough level of 0.5–1.0 mg/L may be optimal [4]. In our patients, we started ganciclovir therapy at 5 mg/kg per dose every 12 h. The ganciclovir peak level sufficiently increased; therefore, a ganciclovir dose of 5 mg/kg per dose appeared to be optimal. In contrast, the trough levels varied with the drug interval, her age, and her condition, making it difficult to set up the optimal treatment plan.

Side effects of ganciclovir include neutropenia, hearing loss, bone marrow suppression, raised liver enzymes, hypokalemia, and renal impairment [6, 7]. In a previous pharmacokinetic study of the use of intravenous ganciclovir in infants with symptomatic congenital cytomegalovirus, the target AUC_0–12_ was defined as 27 m · h/L to avoid side effects [8]. In our case, thrombocytopenia worsened upon ganciclovir therapy; however, it was not due to ganciclovir, but probably because of hepatocirrhosis with biliary atresia. There was no evidence of other side effects, including neutropenia and renal impairment. Apparent lack of side effects may be due to the fact that we performed the ganciclovir TDM analyses and avoided high AUC accordingly.

We believe that ganciclovir therapy can bring about both virological improvement and clinical benefits, and propose that it should be a therapeutic option for severe postnatal CMV infection in extremely premature infants. The present case had such complicating and serious clinical conditions as extreme prematurity and hepatobiliary dysfunction. Ganciclovir is excreted largely unchanged by the kidneys and hepatic metabolism of the drug is only minimal. Therefore, it is quite likely that pharmacokinetics of ganciclovir was influenced by renal insufficiency and characteristic body fluid composition accompanied by prematurity. While direct influence of hepatobiliary disorder on drug metabolism is unlikely, severe liver failure can cause secondary renal dysfunction, which might influence pharmacokinetics of the drug in this patient. In this regard, we believe TDM is needed in any complicating and serious clinical condition. It is important, therefore, to analyze the ganciclovir TDM and CMV load to secure the efficacy and safety of ganciclovir therapy in extremely premature infants. Since there have been only limited data regarding the pharmacokinetics, safety profiles, and adverse effects of ganciclovir in infants, especially extremely premature infants [9], further studies are warranted to establish antiviral treatment for them.

## Additional file

Additional file 1: Timeline. (PPTX 62 kb)

## Abbreviations

AUC: Area under the concentration–time curve
CMV: Cytomegalovirus
PCR: Polymerase chain reaction
TDM: Therapeutic drug monitoring
VL: Viral load
