# Supplementary material for: Therapeutic drug monitoring of ganciclovir for postnatal cytomegalovirus infection in an extremely low birth weight infant: a case report
Source: BMC Pediatr. 2016 Aug 22;16(1):141. doi: 10.1186/s12887-016-0683-x (PMC4994216; doi:10.1186/s12887-016-0683-x)
Supplement: Additional file 1: — Timeline. (PPTX 62 kb) [file 12887_2016_683_MOESM1_ESM.pptx]

## Slide 1
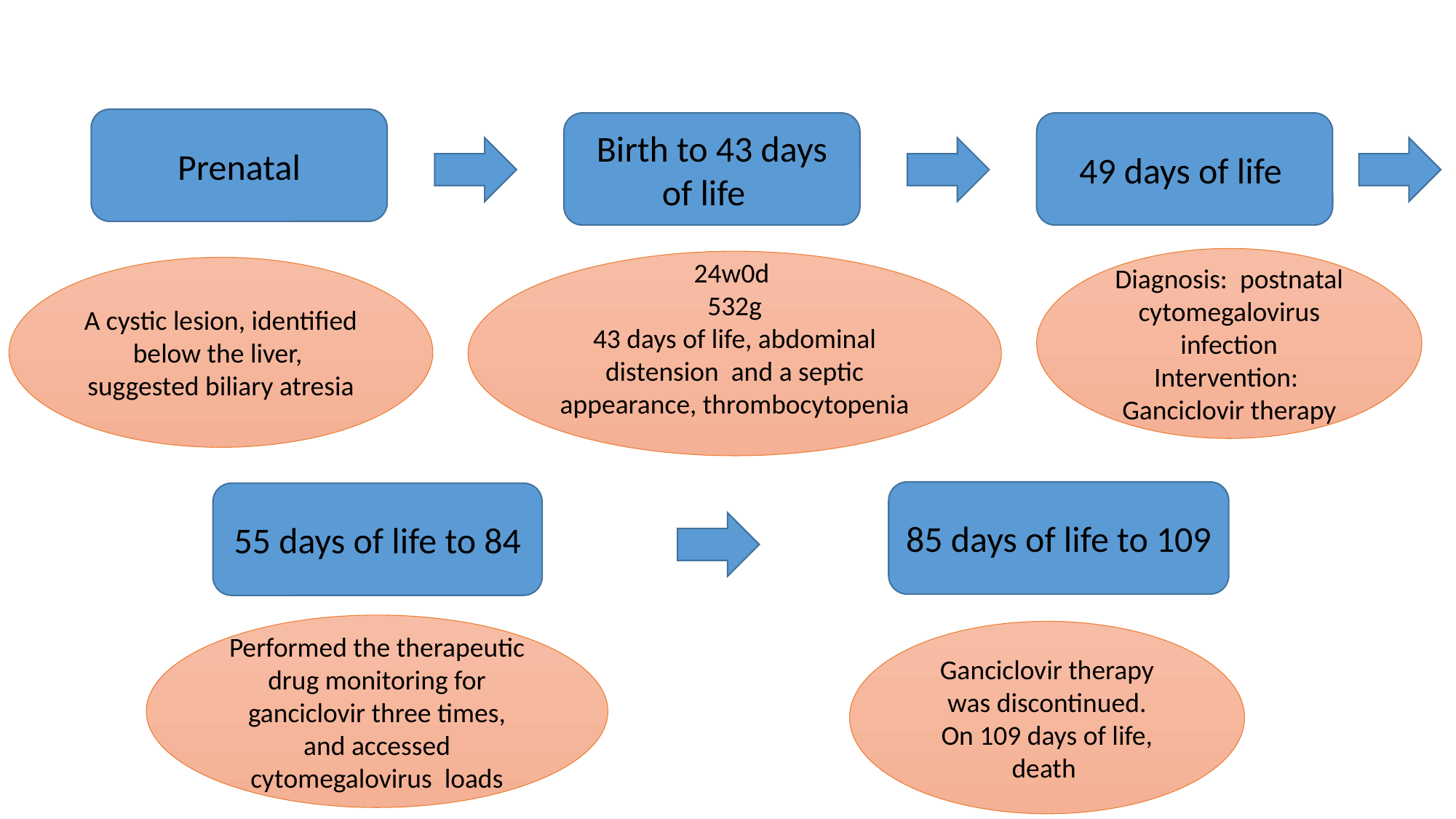

Prenatal
Birth to 43 days of life
49 days of life
Diagnosis: postnatal cytomegalovirus infection
Intervention:
Ganciclovir therapy
24w0d
532g
43 days of life, abdominal distension and a septic appearance, thrombocytopenia
A cystic lesion, identified below the liver,
suggested biliary atresia
85 days of life to 109
55 days of life to 84
Performed the therapeutic drug monitoring for ganciclovir three times, and accessed cytomegalovirus loads
Ganciclovir therapy was discontinued.
On 109 days of life, death
